# Immune Cells and Microbiota Response to Iron Starvation

**DOI:** 10.3389/fmed.2018.00109

**Published:** 2018-04-18

**Authors:** Marcello Chieppa, Gianluigi Giannelli

**Affiliations:** National Institute of Gastroenterology “S. de Bellis”, Research Hospital, Castellana Grotte, Italy

**Keywords:** iron-chelating agents, microbiota, inflammation, inflammatory bowel disease, immune cells

## Abstract

Metal ions are essential for life on Earth, mostly as crucial components of all living organisms; indeed, they are necessary for bioenergetics functions as crucial redox catalysts. Due to the essential role of iron in biological processes, body iron content is finely regulated and is the battlefield of a tug-of-war between the host and the microbiota.

Iron availability in the intestinal lumen could prevent or promote intestinal dysbiosis, although current data do not provide a definitive response. Recent data demonstrated that nutritional derived polyphenols explicit their anti-inflammatory functions sequestrating iron from immune cells. Here, we discuss whether nutritional iron chelators could be able to change the gut microbiota composition and prevent the intestinal dysbiosis associated with intestinal chronic inflammatory syndromes.

Iron is lost by cellular exfoliation and occasional bleeding; it is absorbed from nutritional components. Heme is the most important source of dietary iron, while non-heme iron can be absorbed only in the duodenum and the beginning of the jejunum in pH permissive (acid) conditions. Western diets often contain large quantities of foods characterized by a high heme-iron content like meat, fish, and poultry, and small quantities of non-heme-iron content like vegetables, fruits, and nuts. Furthermore, nutritional substances can affect iron absorption: ascorbic acid is an efficient enhancer of non-heme-iron absorption, *vice versa*, phytic acid is known to be among the major iron absorption inhibitors, and iron-chelating substances like quercetin inhibit its absorption, likely due to loss of chelated-iron solubility.

Iron deficiency is the most common cause of anemia worldwide and one of the most common complications observed in inflammatory bowel disease (IBD) patients due to gastrointestinal hemorrhages. In IBD patients, the guidelines for the management of iron deficiency are not entirely satisfactory because following oral iron supplementation patients sometimes report worsening of the IBD symptoms (1). Interestingly, iron supplemented diets can also show protective effects in dextran sodium sulfate (DSS)-induced colitis models. Constante et al. demonstrated that iron formulation dramatically changed the outcome of the DSS-induced colitis, as oral supplementation with ferrous bisglycinate but not ferric ethylenediaminetetraacetic acid enhanced the beneficial action of probiotics (2).

## How the Immune Cells Respond to the Iron Deficiency?

Iron availability may play a non-redundant role in chronic inflammatory syndromes. It may suppress or promote the inflammatory ability of immune cells, and iron-chelating molecules may play a pivotal role in this mechanism. Iron sequestration is a strategy used by the host to restrict pathogen proliferation. Macrophages play a central role in this process, as they are the most important cell involved in removing senescent red blood cells to recycle iron (erythrophagocytosis). Under inflammatory conditions, macrophages, but also monocytes and dendritic cells (DCs), retain iron through ferritin, an intracellular protein that can bind up to 400 atoms of iron. At the same time, iron export is inhibited due to the cascade of events that is triggered by inflammation-mediated increased levels of hepcidin. Hepcidin binds to the iron-export protein ferroportin and consequently induces its internalization and degradation. The overall result is a decreased level of circulating iron and increased load of cytoplasmic iron in macrophages (3). Like other iron-mediated defense mechanisms described below, this strategy is efficient at hampering bacterial growth but seems counterproductive in the case of intracellular pathogens like *Salmonella*. Indeed, mice characterized by low levels of iron in macrophages, due to mutation in the Hfe gene, better control *Salmonella typhimurium* infection (4).

The observation that quercetin-exposed DCs fail to release inflammatory cytokines when treated with LPS was recently linked to the cellular iron content by Galleggiante et al. (5). By chelating iron in the culture media, quercetin promotes iron efflux from DCs, whose inflammatory ability consequently becomes impaired. Quercetin anti-inflammatory properties disappear when the culture media are supplemented with ferrous sulfate, indicating a direct link between iron chelation and DCs’ inflammatory abilities.

## How the Gut Microbiota Respond to the Iron Starvation?

It has long been known that iron availability is crucial for bacterial growth, and iron deprivation is an efficient strategy to limit bacterial growth. Bactericidal properties of iron-chelating phosvitin contained in eggs were (unknowingly) described by Shakespeare (6) in the third act of King Lear “*I’ll fetch some flax and whites of eggs to apply to his bleeding face*.” More recently, an increased risk of bacterial infections has been observed following the administration of non-physiological amounts of iron and, in particular, an increased virulence of *Escherichia, Klebsiella, Listeria, Neisseria, Pasteurella, Shigella, Salmonella, Vibrio*, and *Yersinia* (7).

When studied using murine models of colitis, the increased oxidative stress was identified as the major cause of disease exacerbation following oral iron administration, but several other mechanisms may be important, including endoplasmic reticulum stress, a microbial community shift and immune cells activation. Furthermore, *in vitro* results obtained using the intestinal fermentation model described by Cinquin et al. (8) demonstrated a direct link between iron restricted growth condition and the growth advantage obtained by *Enterobacteriaceae* and lactobacilli (9). Nonetheless, these *in vitro* results were in contrast with Dostal et al. who observed marginal changes in gut microbiota composition in rats under low luminal Fe concentrations (10). A likely explanation for the contrasting results obtained by Dostal et al. is the experimental model used was not Fe deficient, thus, in non-anemic patients, the host Fe reservoir may be sufficient to sustain the healthy composition of the gut microbiota.

## Does Nutritional Iron Implementation Influence the Microbiota Composition?

The relation between iron availability and intestinal microbiota is still largely unexplored although it is well known that iron availability influences the composition of the microbiota. The battle for iron is mainly based on iron-sequestration strategies. From the microbial side, iron uptake relies on iron chelation, high-affinity proteins (siderophores) being a mechanism serving to scavenge this metal from host protein and/or other microbial species. The best-known siderophore is enterobactin, first isolated in 1970 and primarily found in Gram-negative bacteria like *S. typhimurium*. From the host side, the siderophores sequestering protein lipocalin-2 released into the intestinal lumen are an innate defense system serving to limit microbial growth. Nonetheless, the same strategy protecting from bacterial proliferation during healthy periods (lipocalin-2 siderophores sequestration) offers advantages to pathogens that acquire iron through modified siderophores (like salmochelin) which are not recognized by lipocalin-2. Raffatellu et al. demonstrated this concept very elegantly, showing that both WT *Salmonella enterica* and the ionN mutant strain (unable to utilize salmochelin) are able to grow in mice intestinal lumen, but the latter is not able to gain advantages during intestinal inflammation. Furthermore, ionN mutant and WT *S. enterica* strains grow equally well in the inflamed intestine of lipocalin-2-deficient mice (11). Heme-derived iron is an important source of iron for both the host and the intestinal microorganisms. Pathogenic strains grow particularly well in heme-rich conditions due to their efficiency in capturing heme. As demonstrated by Constante et al. in mice, a heme-rich diet decreased microbial diversity, increased the abundance of *Proteobacteria* and reduced the abundance of *Firmicutes* similarly (but to a lesser extent) to DSS-induced colitis (2). Furthermore, a heme-enriched intestinal lumen (due to a heme-rich diet or intestinal bleeding) may favor the growth of bacteria-coding genes related to heme uptake and release from red blood cells. This aspect may be crucial to explain the correlation between meat consumption and increased risks for colorectal cancer.

## Are Nutritional Iron Chelators Able to Change the Gut Microbiota Composition?

As nutrition-derived iron is a crucial aspect of the intestinal ecology, nutrition-derived iron chelators may play an equally relevant role in shaping the microbial composition of the intestine. Direct studies addressing this complex subject are still lacking, but the effects of some iron chelators have been reported. As mentioned earlier, egg white (EWH) is one of the first iron chelators ever described. The non-heme-iron binding pepsin hydrolyzate of EWH was used to supplement obese Zucker rats and evaluate the microbiota modulation. EWH supplementation was able to drive the microbiological characteristics of the obese Zucker rats toward that of the lean rats (12). Polyphenols, characterized by well-known iron-chelating abilities, were reported as antimicrobial agents (13), but there are no direct studies exploring whether polyphenol-mediated effects on the gut microbial composition are directly related to iron sequestration, or else iron-sequestration results in immune cells anti-inflammatory polarization thus influencing the gut microbial composition. Iron sequestration by iron–polyphenol complexes could be an effective strategy to deprive gut microbial species of a crucial supply. Indeed, it is known that the iron–polyphenol complex cannot be absorbed by the epithelial cells and is excreted in the feces (14), suggesting that intestinal bacteria also fail to obtain iron once it has been chelated by polyphenols.

**Figure 1:**
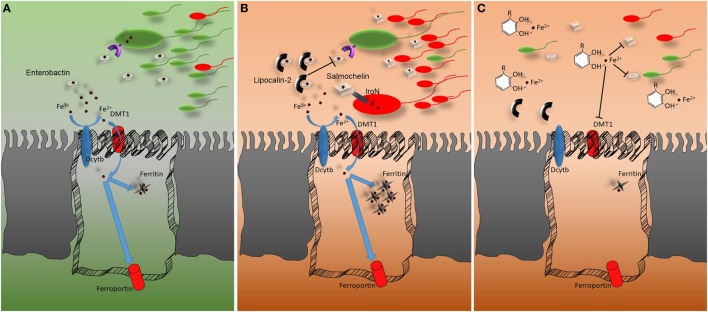
Representation of the host–microbiota battle for iron. **(A)** In homeostatic conditions, Fe^3+^ is reduced to Fe^2+^ by the ferrireductase (DcytB) activity before being imported through the epithelial divalent metal transporter DMT1. Once inside the cells, iron can be accumulated, bound by ferritin, or exported *via* ferroportin. The gut microbiota relies on low-molecular-weight iron chelators (siderophores) for receptor-mediated iron uptake. The most common siderophore is enterobactin. **(B)** In conditions of intestinal inflammation lipocalin-2 secretion from epithelial and myeloid cells is upregulated. Lipocalin-2 reduces iron availability for gut microbiota by binding enterobactin and thus impairing enterobactin-mediated iron acquisition. Pathogens that do not strictly rely on enterobactin for iron uptake gain an advantage from lipocalin-2 mediated commensal growth, which hampers secreting glucosylated variants of enterobactin which are not bound by lipocalin-2. **(C)** Polyphenols can bind iron with strong affinity. DMT1 fails to transport the polyphenol–iron complex into epithelial cells. Most likely, the polyphenol–iron complex reduces iron availability in the intestinal lumen, impairing gut microbiota growth. This aspect should be taken into account during intestinal inflammatory events associated with microbial dysbiosis.

In light of the crucial role of the microbiota in IBD, future studies need to take into account the possibility of using natural iron-chelating agents to suppress immune cells inflammatory responses and, at the same time, to prevent undesired gut microbial advantages (15, 16) (Figure 1). Recent metagenomics studies in diet-induced obesity murine models demonstrated a marked increase presence of *Akkermansia muciniphila* in the intestinal microbiota of mice daily gavaged with cranberry extract. *A. muciniphila*-derived extracellular vesicles seem to be able to improve tight junction expression and improve intestinal barrier integrity. These results provide a link between polyphenol exposure, intestinal microbiota, and intestinal health, but much more has to be done to comprehend the exact ongoing mechanisms (17–20). The infusion route should be preferentially adopted for patients’ needs of iron supplementation to avoid conferring undesired advantages to intestinal pathogens. Nutrition and nutrition-derived therapies should be considered as pivotal complementary treatments that can have an impact on the mucosal immune response. These insights can be viewed from several different perspectives and may help to improve the efficiency of current pharmacological approaches.

## Author Contributions

MC wrote the manuscript. GG edited the manuscript.

## Conflict of Interest Statement

The authors declare that the research was conducted in the absence of any commercial or financial relationships that could be construed as a potential conflict of interest.
